# Analysis of Driving Factors in the Intention to Use the Virtual Nursing Home for the Elderly: A Modified UTAUT Model in the Chinese Context

**DOI:** 10.3390/healthcare11162329

**Published:** 2023-08-17

**Authors:** Zongwei Ren, Guangmin Zhou

**Affiliations:** School of Management, Harbin University of Commerce, Harbin 150028, China

**Keywords:** elderly care, m-Health, virtual nursing home, UTAUT, behavioral intention

## Abstract

As a unique form of mobile health service (m-Health) for elderly care in China, the virtual nursing home is considered a reliable alternative to the traditional model of home-based care, but services from virtual nursing homes are infrequently used by the elderly in practice. Thus, this study aims to measure the driving factors affecting the behavioral intention to use the virtual nursing home by designing a research framework that combined the unified theory of acceptance and use of technology (UTAUT) with the technology acceptance model (TAM). Furthermore, a new variable, conformist mentality, is proposed as a moderator. In order to validate the model, a well-structured questionnaire using mature scales was used, and the linear regression analysis method was conducted on 200 valid data samples collected during a field study in Harbin, China. The results show that performance expectancy, effort expectancy, and social influence significantly affect behavioral intention. However, the effect of facilitating conditions is not significant. Moreover, performance expectancy and effort expectancy have a positive effect on attitude toward use, and attitude toward use not only affects behavioral intention but also plays a mediating role in the effect of performance expectancy and effort expectancy on behavioral intention. This study also innovatively proposes and confirms conformist mentality as a moderator to strengthen the driving effect of social influence on behavioral intention. This is the first time that conformist mentality is introduced as a moderator in a study on the behavioral perception and acceptance of virtual nursing homes among Chinese older adults. Based on these findings, this study offers theoretical contributions and management implications that are conducive to the sustainable development of virtual nursing homes, thereby making extensive contributions to this field. Additionally, it also aids in the contextual expansion of the UTAUT model.

## 1. Introduction

**Figure 1:**
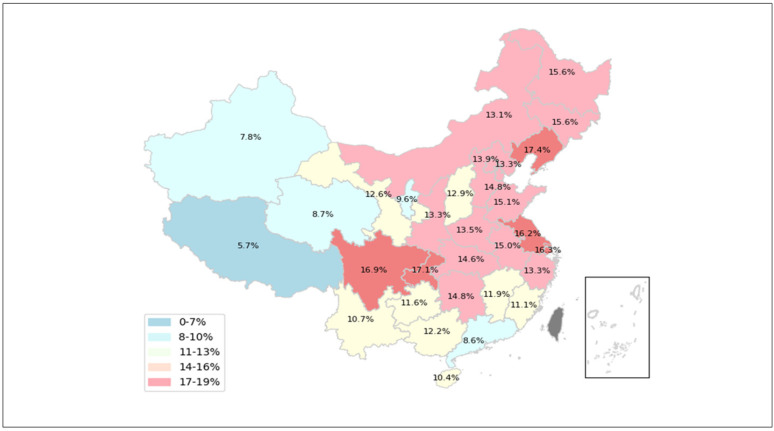
Percentage of the population aged 65 or older in 2020 by location (except Hong Kong, Macau, and Taiwan).

Technological advances have remarkably extended life expectancy, yet inevitably birthed the problem of population aging. Aging is a global issue, and in the face of its challenges, the importance of utilizing gerontechnology to create an accessible environment to enhance health conditions has become increasingly apparent. Studies suggest that technology has the potential for improving life quality for the elderly, especially within smart cities. This includes collaborative models involving public agents and volunteers and intelligent models for accommodating older adults with disabilities in smart city environments [1,2]. Furthermore, technologies like LoRa and MQTT have been explored for older adult monitoring to prevent accidents [3]. Importantly, privacy needs to be protected in all these processes. This is particularly crucial for elderly users, for whom human-centered AI approaches have been proposed to safeguard privacy while reducing cognitive load [4]. It is worth noting that similar to other countries around the world, China has also explored the path of using gerontechnology after entering into the aging stage. However, China, in particular, faces unique characteristics of population aging due to its fertility policy [5]. This phenomenon is particularly evident in China’s population structure, where the dual impact of fewer children and aging has led to a pyramid-shaped population structure in China [6,7]. According to statistics, by the end of 2022, 210 million Chinese citizens were aged 65 or older, comprising 14.9% of the population [8]. These statistics, as depicted in China’s seventh population census data (see Figure 1), highlight the national problem: Aging is an unavoidable fact, and it poses significant challenges for China such as a shortage of working-age labor and a shortage of pensions. Recognizing this, the National Health Commission of China further reiterated the gravity of the situation, stating that the aging crisis will continue to escalate in the next decade, by which time at least 400 million people will face the aging crisis [9]. Indeed, in addition to fertility policies, urbanization and economic pressures are also accelerating the development of this process [5,10]. These factors limited the average Chinese family size to 2.62 persons in 2020 [11], which means that intergenerational support from families will be less viable for the elderly. Despite these challenges, influenced by traditional Chinese culture, the elderly are generally more reluctant to live in institutions. Therefore, in response to this cultural preference and the growing need for elderly care, the Chinese government has implemented a model of elderly care called the “virtual nursing home”, which is based on the needs of the elderly at home combined with modern technology [12].

As an upgraded version of the home care model, the virtual nursing home is home-based and relies on communities. It secures social support with enterprise involvement, utilizing a high-quality online platform as a medium, and integrating several companies to provide services (e.g., housekeeping, medical, and catering). Moreover, by utilizing data collected from smart devices, it can offer personalized health advice to the elderly, taking advantage of the value derived from information and technological advancements. This model transcends original community barriers in reality and establishes an “online health community” with a huge scale and information using the digital network system [13]. In this community, seniors receive professional care in a familiar home environment, promoting “aging in place”. Additionally, they can enrich their spiritual life and strengthen societal connections by participating in a “the university of the third age”. More specifically, the virtual nursing home model integrates intelligent devices, online platforms, and offline networks to address the service requirements of elderly individuals [14]. The platform processes real-time device data, assigning requests to suitable service or product providers. Offline providers fulfill these services while the platform logs work orders, forming a closed-loop, traceable supply chain. 

Currently, the “Ju Jia Le” platform in Suzhou, China’s pioneer in this domain, has been serving a cumulative 3,265,000 elderly individuals over 15 years and providing over 20 million services; the model’s superiority is manifest [15]. Despite the numerous benefits of this model, a significant disparity persists between the number of users and expected demand when replicating and promoting such platforms in various regions. For instance, Harbin, China, established the “Longjiang Smart Senior Care Platform” to create a nursing home without walls by leveraging smart devices as infrastructure. Nevertheless, data from 2021 indicate that, of the 897 communities encompassed by the platform, only 5% availed of the service [16]. While these statistics are region-specific, Harbin, an early industrializing city in northern China, is experiencing a more pronounced aging trend. Thus, it is plausible to hypothesize that analogous situations may be prevalent in other northeastern China regions [17]. 

Such findings underscore that the issue of low service utilization necessitates a further review. Also, in the context of platform-based bilateral network effects, the interdependence between users and service providers highlights the importance of active participation from the elderly population [18]. This engagement can generate increased interest and commitment from service providers, enabling platforms to acquire sufficient resources for dynamic adjustments and improved service quality. Consequently, this enhances the user experience and fosters a more robust and sustainable service ecosystem. Thus, it is vital to explore the factors that drive the intention to use virtual nursing homes and the relationship between these factors. 

To determine which factors contribute to this phenomenon, the following questions were addressed:

Q1: What factors drive the willingness of elderly people to use the service?

Q2: What are the relationships between those factors? 

Methodologically, the unified theory of acceptance and use of technology (UTAUT) model can be used to answer the above questions. It has been widely used as a measurement tool to study willingness to use and behavior in the fields of smart homes, telemedicine, and e-Health [19,20,21]. Along this line of research, previous studies focus on exploring the intention to adopt new technologies among different populations using the UTAUT model. For instance, one investigation examines the utilization of radio frequency identification (RFID) among hospital doctors and nurses [22], while another study from Canada provides empirical support for patients’ intentions to use an emergency department (ED) wait times website [23]. However, specialized research on the elderly remains limited. It is widely accepted that technological innovations and the use of digital devices are primarily directed toward the younger generation rather than the elderly [24]. For example, a survey on the key factors influencing mHealth usage in Bangladesh focuses on Generation Y (18–40) [25]. Nevertheless, during the COVID-19 pandemic, the usage of mobile technologies by the elderly in a home environment gained increasing attention [26]. Although some studies have explored the usage behavior of the elderly, there remains a lack of practical exploration regarding the Chinese context. 

To address these gaps, this study aims to explore the core factors influencing the adoption of mHealth among the elderly in Harbin, China. The virtual nursing home model is a smart nursing product with Chinese characteristics that is affiliated with the scope of healthcare. Therefore, this study uses the integrated UTAUT model as a framework to answer the above questions by putting forward ten hypotheses and verifying them. This study has a dual contribution. Firstly, by conducting an in-depth investigation into the determinants of the elderly’s intention to use virtual nursing homes, we predict their intentions and better meet their needs and expectations. This will promote the sustainable development of virtual nursing homes and provide a higher quality of care and services to the elderly. Secondly, by using the UTAUT model to examine virtual nursing homes in the Chinese context, we extend the scope of the UTAUT model’s application, enriching its theoretical relevance. In conclusion, by analyzing these issues, we increase our understanding of the behavioral motivation of the elderly and propose suggestions for the sustainability of virtual nursing homes. This will help improve the elderly’s adoption of the virtual nursing home to support policymaking in line with current government initiatives. Below, we outline the arrangement of specific details presented in this study.

The rest of this work proceeds as follows: Section 2 describes a literature review of virtual nursing homes and the UTAUT model, and Section 3 presents the conceptual model underpinning this study and hypotheses development. Section 4 introduces the research method, after which the results of the empirical analysis and hypothesis testing are presented in Section 5. Section 6 contains the discussion, including implications, limitations, and future research directions. Finally, Section 7 concludes this article with a summary.

## 2. Literature Review

### 2.1. Virtual Nursing Home

The virtual nursing home is a novel model created in China, drawn on the concept of a “Smart City” [27]. The term “virtual nursing home” was formally introduced in 2007 when China started to explore “digital aging” [28]. Although there is currently no precise definition, based on an analysis of relevant concepts, it is defined as a service platform built by the government or commissioned enterprises integrating multiple franchisees. Using the digital platform, the platform operators achieve precise coordination of the service content [12,14,17]. In recent years, along with the rapid development of information technology, research on the “virtual nursing home” has gained increasing attention from scholars. 

The existing literature on virtual nursing homes and similar structures focuses on three aspects. Firstly, regarding service content, Ozkan et al. argue that smart home care uses interactive functions between the elderly and intelligent devices to support their daily lives [29]. According to Mu et al., the virtual nursing home model uses membership-based management based on modern technology, which consolidates information on the elderly in the region, analyzes needs, constructs an effective service system, and delivers home services through enterprises [30]. Similarly, Li compared two typical practice models of virtual nursing homes and emphasized a four-part structure, which includes a call center client, an elderly home client, a platform service component, and an information transmission system. These subsystems ensure the effective utilization of six functions: work order generation, process management, service monitoring, charge inquiry, statistical analysis, and demand prediction. Based on these functions, the platform builds a service system that combines medical care and health care [14,15,31]. After examining disparities in research among scholars, it became clear that the primary emphasis of smart home care is on the integration of intelligence and automation within the residential context, with a particular focus on the interactive capabilities of smart devices [32]. This approach aims to help the elderly regain independence with the implementation of an ambient assisted living system (AAL) [33]. In contrast, virtual nursing homes represent a comprehensive and integrated platform that goes beyond smart home technology. It encompasses a wide range of services, including personal care, healthcare, social engagement, and smart home assistance, to meet diverse needs while strengthening social connections [12]. Secondly, regarding the analysis of user needs, Ismail et al. administered a telemedicine satisfaction survey and discovered that elderly individuals who aged at home had higher telemedicine satisfaction than those living in nursing homes [34]. In addition, Bai et al. found different levels of need in catering, cleaning assistance, and remote care using a study of intelligent home care users in Wuhan [35]. Thirdly, in terms of practical exploration, Helal et al. developed an intelligent home furnishing system called “Gator Tech” to smarten the home environment using an ultrasonic positioning system to track users’ mobility habits and adjust their home layouts [36]. Tai constructed a dynamic health cloud elderly care service application platform to meet the urgent requirements of the elderly population, integrating traditional Chinese medicine wisdom with big data and providing real-time and effective healthcare services to older users [37].

### 2.2. Unified Theory of Acceptance and Use of Technology

**Figure 2:**
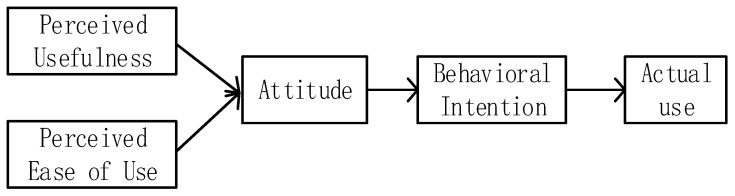
The technology acceptance model (Davis 1986 [39]).

**Figure 3:**
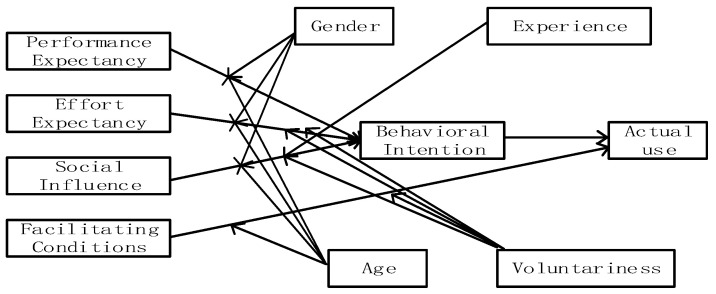
The unified theory of acceptance and use of technology (Venkatesh et al., 2003 [38]).

The unified theory of acceptance and use of technology (UTAUT) was first proposed by Venkatesh et al. [38], who expanded on the technology acceptance model (TAM) by integrating eight previously existing theoretical models that were used to discuss studies related to users’ usage behavior. The TAM model (see Figure 2), proposed by Davis in 1986 [39], includes two important variables: perceived usefulness (PU) and perceived ease of use (PEOU), which represent the extent to which individuals believe that using the new technology will be useful for their current work and the extent to which individuals feel that the new technology is easy to operate during the process of use, respectively [39]. These two variables affect user attitude. Attitudes not only influence the user’s intention to use the new technology or systems but also influence behavior through intention. Building on the foundation of TAM, UTAUT formed four core variables, namely, performance expectancy (PE), effort expectancy (EE), social influence (SI), and facilitating conditions (FCs), and a dependent variable called usage intention (see Figure 3) [40,41]. PE and EE in UTAUT can be seen as an extension and refinement of PU and PEOU in TAM, while SI and FC are new factors introduced in UTAUT, making it more comprehensive and in-depth in explaining technology acceptance and usage behavior. With the rapid development of information technology, promoting users’ willingness to use and behave is crucial for the survival and development of new technology. UTAUT, along with related models, such as TAM and TPB, serves as the foundation for studying user acceptance and use of new technologies in the Internet+ environment, including telemedicine, smart devices, and mobile payments.

Cimperman et al. [19] expanded the UTAUT model in their study of HTS, introducing computer anxiety and physician opinion. Their findings showed these variables, except for social influence, significantly influenced elderly users’ HTS acceptance. Yamin and Alyoubi delved into the adoption of telemedicine applications and asserted that task technology matching, awareness, and self-efficacy, along with the foundational variables in the UTAUT model, collectively influenced the intention to use wireless sensor network applications (WSN) [20]. Maswadi et al. conducted a study in Saudi Arabia that investigated the utilization of smart homes among older adults using the UTAUT model, and their results confirmed the moderating effects of region and education [42]. Likewise, Alkhowaiter used a cross-sectional survey, leveraging a meta-UTAUT model, to conduct an in-depth examination of the role of Islamic religiosity as a moderating variable in mobile payment behavior within Gulf countries [43]. After analyzing previous studies, we found that current research on the UTAUT model mainly focuses on two aspects: The first direction focuses on individual psychological characteristics, while the second direction takes into account group environments and cultural backgrounds. Each direction adjusts the model structure by integrating other theories or adding new variables, and using empirical analysis, explores the usage intention and behavior of “Internet+” service users.

### 2.3. Limitations of Previous Studies

In summary, scholars generally recognize the enormous potential of virtual nursing homes to complement or even replace traditional models of aging. However, the problem is that current research focuses on the macro level and lacks further refined empirical studies. Meanwhile, as listed in Table 1, concerning the UTAUT model, the existing literature has separately explored the intention to adopt digital technology from the perspectives of cultural backgrounds and psychological characteristics [19,20,42,43,44,45,46], with limited attention to the combined usage of these factors. Therefore, there is a specific research gap in understanding the intention to use virtual nursing homes among the elderly in the Chinese context. To address this gap and advance theoretical research, it is necessary to further investigate the key factors driving the intention to use virtual nursing homes among the elderly population in China. The next section will mainly focus on refining variables and adjusting the UTAUT model to incorporate core factors influencing the intention of elderly individuals to use virtual nursing home platforms, considering the Chinese context and personal characteristics. This will ensure consistency with the research topic.

## 3. Research Model and Hypotheses

The UTAUT model is not always applicable to all situations, and proper modification of the model is required. This study aimed to analyze the driving factors that influence behavioral intention to adopt virtual nursing homes rather than actual usage behavior. Some studies demonstrated a direct relationship between facilitating conditions and behavioral intention [47,48], so this study uses the four core drivers of performance expectancy, effort expectancy, social influence, and facilitating conditions as independent variables; behavioral intention is the dependent variable. Secondly, the virtual nursing home, as an inclusive elderly care model, has specially designed call center services for users who are not familiar with the Internet or smartphones. With just one touch of a button, users can enjoy a wide range of convenient and efficient services [31]. This design reduces the elderly’s dependence on their experience in using smart devices. Therefore, when evaluating user acceptance of virtual nursing home platforms, we believe that the “experience” variable is no longer a significant factor, leading us to remove it from the model. In addition, the government has explicitly given the elderly the freedom to choose their aging method (such as institutionalized aging, travel aging, self-aging, etc.) and prohibited the forced use of smart devices by the elderly [49]. Thus, this study takes the position that elderly people voluntarily generate an intention to use and removes “voluntariness” [50]. 

**Figure 4:**
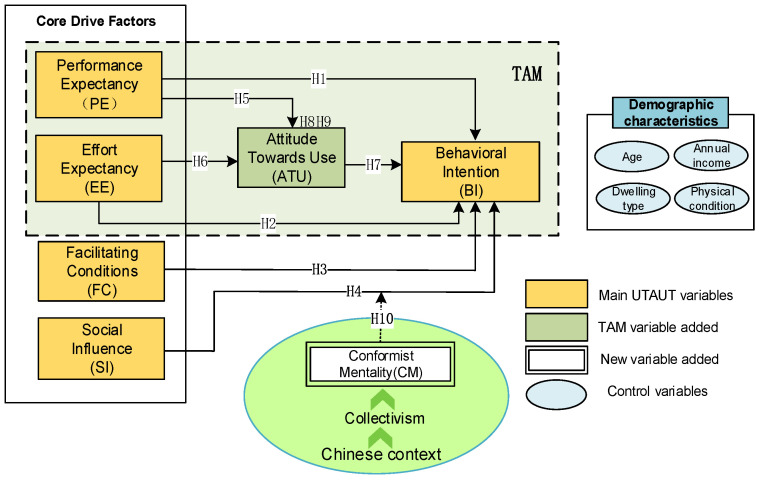
Driving force model for the willingness to use a virtual nursing home.

In this study, “attitude” initially is defined as a psychological orientation toward a specific object or event, either positive or negative, manifesting as a crucial individual psychological characteristic [41]. Based on this, we integrate “Attitude Towards Use” (ATU) as a mediating variable, influencing the impact of PE and EE on BI. This integration considers the significance of perceived usefulness, perceived ease of use, and usage intention in the TAM [51]. When particularly focusing on the cultural context of China, we further incorporate “Conformist Mentality” (CM), a psychological phenomenon under a collectivistic background, into the model [52]. Certain research indicates that conformity psychology impacts behavioral intention not directly but through moderating other factors and consequently influencing intention [53,54]. Therefore, this study sets CM as a moderating variable, aiming to investigate how it strengthens social influence, thereby driving the usage intention of elderly users. Finally, although control variables are not the focus of this study, they will undeniably influence the analysis results; therefore, four demographic characteristics, including age, dwelling type, physical condition, and income, were added to the model as control variables. Figure 4 shows the conceptual model built in this study. The following section will discuss the development of the hypotheses based on these predictors. 

### 3.1. Hypothesis Formulation Based on the UTAUT Model

Performance expectancy is a crucial variable in the UTAUT model [38], for example, Zhou et al. argue that users will find the system useful and adopt it when they believe that the new technology will meet their needs and enhance their performance [55]. Given the focus of this study on the “virtual nursing home” service platform within the health domain, performance expectancy (PE) refers to the perception of the elderly that the use of virtual nursing home service platforms will help them to better enjoy the elderly services and more effectively meet the needs of the elderly population [56,57]. When they have greater PE, the elderly’s willingness to use virtual nursing homes is predicted to increase. Accordingly, the following hypothesis is proposed:

**Hypothesis 1 (H1).**  *PE will have a significant positive driving effect on BI*.

Effort expectancy is expressed as the degree of effort required for users to accept a new technology service [38], and components such as Perceived ease of use (TAM), and system complexity (MPCU) are included within this concept. In this study, effort expectancy (EE) refers to the perceived ease of operating virtual nursing home platforms by the elderly, i.e., whether the interface settings are clear and whether the operation methods are simple. The higher the effort expectations, the more willing the older adults are to use the platform. In recent years, the UTAUT model has become widely used in the field of public management, and scholars believe that the effort expectancy of new technologies affects individual willingness to use them [58]. Thus, the following hypothesis is postulated:

**Hypothesis 2 (H2).**  *EE will have a significant positive driving effect on BI*.

Facilitating conditions are the extent to which users perceive that the organization can support the use of new technology services. For the UTAUT model [38], Kijsanayotin underscored facilitating conditions as being an important factor affecting user adoption of health information technology, with both organizational support and technical support being important facilitators of user adoption [59]. In this study, FC refers to the extent to which older adults believe that the existing technological conditions can support their use of virtual nursing home platforms, i.e., older adults believe that the platform provides them with an effective feedback mechanism. However, if they frequently encounter unsolvable operational issues, the likelihood of discontinuing the platform’s use increases; hence, hypothesis 3 is postulated:

**Hypothesis 3 (H3).**  *FC will have a significant positive driving effect on BI*.

Social influence is defined as the degree to which individuals perceive that important people agree with their specific behaviors [38]. The reaction and feedback from the external environment to novel systems can serve as a basis for user decisions, and existing research suggests that social influence makes a significant contribution to the effect of willingness [60]. Given that the concept of virtual nursing homes has yet to gain widespread acceptance in society, endorsement and promotion by the government and other authoritative institutions are particularly critical in enhancing the willingness of the elderly to use it. Hence, within the context of this study, social influence (SI) refers to the extent to which friends, relatives, and authoritative entities like the government recommend elderly use of virtual nursing homes. Based on this understanding, the following hypothesis is asserted:

**Hypothesis 4 (H4).**  *SI will have a significant positive driving effect on BI*.

### 3.2. Hypothesis Formulation Based on the TAM

Attitude toward use is viewed as a user’s positive or negative feelings toward a particular behavior. This attitude significantly impacts whether a new technology will be adopted. In TAM theory, attitude is influenced by perceived usefulness and perceived ease of use, which, respectively, represent an individual’s evaluation of a new technology’s applicability to their current work and the ease with which they can use the technology [38]. The concepts of PE and EE are aligned with these ideas. PE incorporates perceived usefulness, external stimulation, and professional applicability, whereas EE encapsulates perceived ease of use, operational simplicity, and system complexity. Although an attitude variable was not mentioned in the original UTAUT, in subsequent studies about the mobile communication domain, researchers emphasized that PE and EE significantly influence attitudes [61]. Dwivedi et al., after expanding the UTAUT model into a meta-UTAUT framework, also confirmed that PE and EE are important variables influencing attitudes in the information systems environment [62]. Therefore, the following hypotheses are formulated by combining the above discussion:

**Hypothesis 5 (H5).**  *PE will have a positive driving effect on ATU*.

**Hypothesis 6 (H6).**  *EE will have a positive driving effect on ATU*.

**Hypothesis 7 (H7).**  *ATU will have a significant positive driving effect on BI*.

### 3.3. The Mediator’s Role in Attitude toward Use

As inferred above, it was found that PE and EE have different effects on the willingness of older adults to use a service as their attitudes change [61,62]. This dynamic reveals a complex psychological mechanism by which individual drivers translate into a willingness to use. Faced with the option of a virtual senior care service, older adults evaluate what benefits the system will bring to them and thus determine whether they will be willing to use it. In this process, the assessment of PE and EE will indirectly impact BI depending on whether the attitude is positive or not. Therefore, the following hypotheses are made:

**Hypothesis 8 (H8).**  *ATU will mediate the driving effect of PE on BI*.

**Hypothesis 9 (H9).**  *ATU will mediate the driving effect of EE on BI*.

### 3.4. Moderating Effects of the Conformist Mentality under Collectivism

The conformist mentality is one of the concrete symbols of collectivist tendencies in the Chinese context [52,63]. The conformist mentality (CM) has significantly influenced user behavior in many domains [64,65,66,67]. This mentality is a reflection of an individual’s acceptance of external social pressure, which is influenced by different cultural environments. Social identity theory states that under the influence of collective values, people are more concerned with the views and opinions of their own group [68]. In the context of China, a substantial portion of the current elderly population experienced their formative years in the unit system society before the advent of the reform and opening up, thereby forging a deep-seated personal bond with their respective units [69]. The influence of this early environment extends into their retirement years, promoting respect for authority and a propensity for group affiliation. This heightened sense of social belonging fosters a conformist mentality within this demographic. In this study, the virtual nursing home platform is assumed to have cultivated a boundary-less virtual community. As surrounding older adults join the community, others, influenced by the conformist mentality and a sense of belonging, develop a herd mentality. This dynamic amplifies the influence of others’ opinions on their behavior, which can be interpreted as “I am willing to try new things to keep up with others, but when those around me resist new things, my willingness to accept new things diminishes.” In essence, the rule-following mentality intensifies the driving effect of social influence on the willingness to use, thereby bolstering the willingness to use. As a result, Hypothesis 10 was formulated.

**Hypothesis 10 (H10).**  *In the cultural context of Chinese collectivism, CM will moderate the driving effect of SI on BI*.

## 4. Research Methodology

### 4.1. Questionnaire and Measurement

This study used a questionnaire to collect data. The questionnaire consisted of three parts. Part 1 was a brief introduction to the questionnaire. It provided the elderly with general knowledge of the survey content to facilitate their responses. Part 2 collected demographic characteristics, including gender, age, residential status, etc. Part 3 was the main part of this study, i.e., the survey on the driving force of the elderly’s behavioral intention to use virtual nursing homes. The item design was appropriately modified to the subject matter of this study after referring to the maturity scales used in existing studies. We used a 5-point Likert scale, with 1 denoting “Strongly disagree” and 5 “Strongly agree”, to construct a questionnaire containing seven-dimensional components (see Appendix A). These components were derived from various studies and included: performance expectancy (PE) was derived from Chen et al. [70], effort expectancy (EE) and facilitating conditions (FCs) were adopted from Venkatesh et al. and Koufaris et al. [38,71], social influence (SI) was adopted from Kijsanayotin et al. and Koufaris et al. [59,71], attitude toward use (ATU) from Schierz et al. [72]. Additionally, conformist mentality (CM) was adopted and modified by Sun, Park et al. and Song et al [65,73,74]. Lastly, behavioral intention (BI) was derived from Warkentin et al. [75].

To guarantee the readability of the content of the questionnaire, experts in the field were invited to suggest modifications to the wording of the statements, thus forming the initial questionnaire. Subsequently, a researcher in our group conducted a pre-survey to guarantee the validity of the questionnaire items, limited to virtual nursing homes as a novel nursing model, for which the public has a low degree of awareness. Thus, this study chose the Nangang District of Harbin in China, which is equipped with a smart nursing service platform called “Jia Le Hui”, as the survey area. The elderly in this area have a certain understanding of virtual nursing homes as the area was coordinated with a guide center of intelligent nursing services, and an urban area town (street), a community tertiary home nursing system, was constructed, where the safety of elderly was protected with new technologies such as infrared. This understanding helped us conduct this study. Researchers in our group administered the questionnaire to 50 older adults residing in the “FanRong” Community on Garden Street, Nangang District, Harbin, China, and the responses were collated to obtain 37 valid questionnaires. According to the verification, the data from the pre-survey had good internal consistency (Cronbach’s α > 0.8, KMO > 0.8), so formal data collection was performed.

### 4.2. Data Collection

During the formal survey stage, a wide range of surveys were conducted on the streets where the “FanRong” Community is located. Considering the elderly’s unfamiliarity with online questionnaires, an offline field survey was selected to ensure their participation. Before conducting the survey, all respondents were informed that the survey data would only be used for research purposes, their anonymity would be guaranteed, and the results would only be reported in an aggregated form. To further motivate them to participate, we offered a post-event gift. However, due to limitations in the respondents’ abilities, there were real-world problems that made it difficult to fill in the survey on their own, so part of the questionnaire was completed by the investigators using face-to-face questions and answers. To ensure the respondents were representative, this study combined the World Health Organization’s definition of the standard age of the elderly. Thus, survey subjects were identified as elderly over 60 years of age who had used a “virtual nursing home” on Garden Street in Nangang District, Harbin. The final questionnaire was handed out to 240 persons, and 217 were returned, yielding a recovery rate of 90.4%. After removing partially invalid questionnaires. A total of 200 valid questionnaires were retained as the sample in this study. Of the sample, 43.0% were male and 57.0% were female. More than half of the participants were in the age range of 60–69 years old (62.5%) and the majority of respondents completed at least high school and beyond (62.2%). The sample results were basically in line with the general situation of the elderly population in Harbin. Detailed socio-demographic characteristics of the responders are listed in Table 2.

## 5. Data Analysis and Results

### 5.1. Validity and Reliability

In this study, two indicators, Cronbach’s α coefficient and combined reliability (CR), were used for reliability testing (see Table 3). The Cronbach’s α coefficient for the total scale is 0.949, and the Cronbach’s α coefficient values for the subscales, ranging from 0.830 to 0.955, are above the standard value of 0.7 [76]. The CR values for all subscales are also above 0.7, indicating that the measurement in this study has high reliability and stability.

The research validity test was divided into content validity and construct validity (convergent validity, discriminant validity). First, for content validity, the choice of items to use in the relevant scale, which was proved by scholars, was revised in combination with the research questions. In addition, the wording was revised repeatedly after expert interviews and the pre-survey to optimize the content, so the research scale has good content validity. Second, for construct validity, the average variance extracted (AVE) for the latent variables and the factor loadings of the observed variables were first used to measure convergent validity. The AVE of each latent variable is higher than 0.5, and the factor loading of each question item is higher than 0.7; therefore, combining these two measures leads to the conclusion that the convergent validity for each latent variable is good. Next, the discriminant validity was checked by contrasting the square root of the AVE values for each latent variable with the Pearson correlation coefficients [77]. As listed in Table 4, the Pearson correlation coefficients for the non-diagonal elements are all less than 0.8, indicating that there is no covariance in the data. Furthermore, the Pearson correlation coefficients are less than the square root of the AVE for the corresponding diagonal elements, and most of the correlation coefficients are around 0.5 in absolute value, meaning that the variable is correlated but not strongly correlated, indicating that the discriminant validity of the overall scale is ideal. Finally, the overall goodness of fit of the model was further examined (see Table 5). The outcome shows a good fit of the model and all indexes; so, it was deemed that the model in this study is acceptable.

### 5.2. Hypothesis Testing

#### 5.2.1. Examination of the Effect of Core Driver Factors on BI

This study conducted a regression analysis of the model using spss22.0 software to verify the proposed hypotheses. To control for the effects of demographic characteristics on the main effects, the control variables were first put into the regression equation, which included four variables: age, physical condition, dwelling type, and income. Then, we added BI into the dependent variable, and finally, PE, EE, SI, and FC were included as the independent variables, and the multivariable linear regression analysis was performed.

As shown in Table 6, Model 1, which only contains the control variables, indicates that the control variables do not significantly affect BI. From Model 2, after controlling for the demographic variables, the standardized regression coefficient for PE (β = 0.169, *p* < 0.01) suggests that performance expectancy has a significant positive effect on the elderly’s behavioral intention to use virtual nursing homes. Thus, H1 is verified. Similarly, effort expectancy (β = 0.334, *p* < 0.001) and social influence (β = 0.318, *p* < 0.001) positively and significantly influence behavioral intention, suggesting that H2 and H4 are supported by the data. However, from Model 6, the effect of facilitating conditions on behavioral intention to use is not significant (β = −0.009, *p* > 0.05), and H3 is thus rejected.

#### 5.2.2. Examination of the Effect of PE and EE on ATU

From Model 6 and Model 7, after excluding the effect of control variables, the standardized regression coefficient of performance expectancy (β = 0.846, *p* < 0.001) indicates that performance expectancy has a positive driving effect on attitude, and thus H5 is supported. The standardized regression coefficient for effort expectancy (β = 0.106, *p* < 0.001) indicates that effort expectancy has a positive driving effect on attitude, and thus H6 is supported.

#### 5.2.3. Examination of the Effect of ATU on BI

From Models 1 and 3, it can be seen that attitude (β = 0.485, *p* < 0.001) has a significant positive effect on behavioral intention to use virtual nursing homes. Thus, H7 is supported.

#### 5.2.4. Examination of the Mediating Effects of ATU

In this study, the mediating role of attitudes was verified using the method of Wen et al. and Baron et al. [78,79]. Although the causal-steps approach has a problem of low inspection ability if the test results of each step are significant, the causal-steps approach is better than the bootstrap method [80,81]. Firstly, after controlling for the demographic variables (Model 6), the significant effects of PE and EE on BI were obtained from Model 4 (β = 0.294, *p* < 0.001; β = 0.459, *p* < 0.001), followed by the significant effects of PE and EE on ATU from Model 7 (β = 0.846, *p* < 0.001; β = 0.106, *p* < 0.001). Finally, the joint effect of PE, EE, and ATU on BI was verified using Model 5. From Models 4 and 5, the standardized regression coefficients for PE decreased from 0.294 to 0.235, the standardized regression coefficient for EE decreased from 0.459 to 0.377, and neither significance changed. In addition, the standardized coefficients for attitude on the mediator variables are significant, so H8 and H9 are supported.

#### 5.2.5. Examination of Moderating Effect

**Figure 5:**
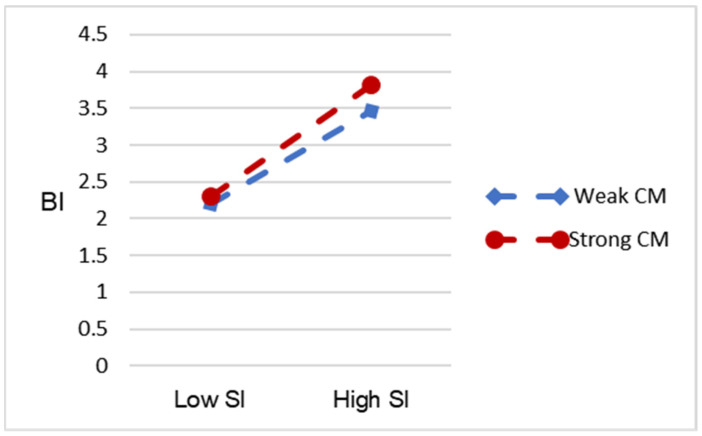
The moderating effect of CM on the relationship between SI and BI.

This study aims to verify the moderating role of conformist mentality between social influence and behavioral intention, drawing reference from the findings of Fan et al. [82]. The results are listed in Table 7, which indicate that the interaction term’s (SI×CM) regression coefficients are significant (β = 0.145, *p* < 0.01). This signifies a moderating effect of conformist mentality on the relationship between social influence and behavioral intention of older adults; thus, H10 is confirmed. Figure 5 was created to visualize the moderating effect of the conformist mentality; it visually presents the impact of social influence on adopting virtual nursing home platform services for older adults with high and low levels of conformist mentality. Specifically, the stronger the conformist mentality of the elderly, the greater the effect of social influence from the outside. This, in turn, strengthens their willingness to use virtual nursing homes. In summary, all the hypotheses are validated with the exception of H3.

## 6. Discussion

This section commences with a comprehensive summary of the results and then discusses them in relation to previous research findings. It then considers the theoretical and practical implications of the results and future research directions.

### 6.1. Principal Findings

The purpose of this study is to explore which driving factors influence behavioral intention to use virtual nursing homes to promote the future development of virtual nursing homes in China. To achieve this, we used a multiple linear regression method to test an integrated model that incorporates the unified theory of acceptance and use of technology (UTAUT) model [38] and the technology acceptance model (TAM) [41] while also considering the impact of the cultural environment [52,69]. Based on this, except for hypothesis H3, which was not supported, all other hypotheses were validated. The following is a specific discussion of the results. 

First, we verified the effect of core drivers in the UTAUT model on behavioral Intention to use by examining H1–H4. This study found that performance expectancy, effort expectancy, and social influence had significant positive driver effects on the behavioral intention of the elderly to use virtual nursing home platforms. The results of this study are consistent with previous research on the use of UTAUT in mHealth, including telemedicine and smart home use [19,20,21,38,48], which concluded that PE, EE, and SI are the core variables influencing willingness to use. Unexpectedly, our study shows that the influence of facilitating conditions on the intention to use virtual nursing homes is not significant. This finding is not the same as some previous studies [25,48]. 

In addition, unlike most studies that exclude the influence of attitudes [38,48,83], the present study reintroduced attitudes after reference to the TAM [41] and meta-UTAUT models [43], which is similar to the concept that “Attitude is a precursor to behavioral intention”, as introduced by Dwivedi et al. [62]. The results for H5 and H6 suggest that performance expectations similar to the concept of perceived usefulness and effort expectations related to perceived ease of use have a significant effect on attitudes. However, in a comparative study, scholars disputed whether attitudes should be included in behavioral adoption studies, and instead, they emphasized the importance of attitudes in voluntary contexts [84]. The role of attitudes is more important in such contexts than in coercive situations where user autonomy is limited. This is highly consistent with the context of this study, in which older adults voluntarily generate a willingness to use smart devices in the context of governmental prohibitions on forcing them to do so [49]. Thus, attitudes have a mediating effect on willingness to use (H7–H9).

Finally, the result for H10 confirms the positive moderation of conformist mentality in the model. Some studies discussed the impact of herd behavior on the behavioral intentions of the elderly as an independent variable [67]. However, our perspective is different. We define conformist mentality as a specific manifestation of collectivist tendencies within the context of Chinese culture, and we believe that its related content plays a moderating role in the model. This viewpoint has been widely accepted in other studies and was confirmed in our tests [85,86].

### 6.2. Theoretical Contributions

This study makes theoretical contributions from the following aspects. First, the “virtual nursing home” is a new type of care and a new technical product in the Chinese context. It can optimize and integrate online and offline resources and information and help improve the current elderly care problem. However, the development of virtual nursing homes is still in the initial stage, and the theoretical research lags behind the practical needs. The current research mainly focuses on the introduction of the concept of virtual nursing homes and the construction technology of information systems [1,30,36,37], but there is less research on the willingness of the elderly to use “Virtual nursing homes”. Therefore, this study enriches the theoretical study of the “virtual nursing home” using empirical analysis. Second, the UTAUT model, as the main research theory exploring the willingness of actors to use, has not yet been applied to the particular issue of virtual nursing homes in the Chinese context. Therefore, we modified the UTAUT based on the characteristics of virtual nursing homes, designed a new scale, and constructed a model for the driving force of the elderly’s willingness to use virtual nursing homes, which expands the application of the UTAUT theory in the field of smart home care. Third, this study focused on examining the role of attitudes in performance expectancy and effort expectancy on behavioral intention. Moreover, the model introduced a new variable, conformist mentality, as a moderating variable and found a new association under the mechanism of action from social influence on the intention to use. Therefore, it is suggested that the effect of a conformist mentality on the elderly should be emphasized in future studies.

### 6.3. Management Implications

This study also has management implications. With the deepening of aging in China, virtual nursing homes promoted by the government will become an inevitable option to improve the quality of elderly care in China against the background of “getting aging before getting rich”. By identifying the driving factors affecting the behavioral intention of virtual nursing homes, we propose management suggestions to optimize the services of virtual nursing homes and improve the usage of virtual nursing home platforms.

(1) Focusing on needs and improving usefulness

In this study, performance expectancy refers to the degree of usefulness of virtual nursing homes as perceived by the elderly, which is based on their subjective perceptions. Therefore, prioritizing the needs of the elderly is of paramount importance before the construction of virtual nursing homes. To achieve this, field research should be conducted to identify the services that are of most interest to seniors in the region. An insufficient survey will lead to low usage of the platform, which may not fully protect the elderly. According to a realistic scenario, such a problem does exist in the current practice process of virtual nursing homes in China, and some regions do not consider regional differences but follow the established models in other regions, resulting in missing construction functions or wasted resources. This means the virtual nursing home should focus on the needs of the elderly, connect the user’s physical condition with the health management system, and realize dynamic supervision of health data. Secondly, the platform should develop a variety of personalized services such as housekeeping services, appliance maintenance, goods delivery, etc., to meet the needs of the elderly in their daily lives. At the same time, the satisfaction of psychological needs is also necessary, e.g., the establishment of a university for the elderly, interest classes, etc. Only when the service items are rationally set up with the needs of the elderly, it will be possible to improve the usage willingness of the service platform.

(2) Building an aging-friendly platform to improve the ease of use

Effort expectancy in this study refers to the ease of use of virtual nursing homes as perceived by the elderly, that is, whether a virtual nursing home is easy to operate. That means the platform’s simple interface, easy-to-use devices, and ready-to-use services will inspire seniors to use it. Using existing Internet software as an example, some service systems do not have a specific service model for the elderly, or their function is only font size adjustment, with minimal support for the elderly, which affects the willingness of the elderly to use a service [87]. Thus, it is crucial to build an aging-friendly service platform and develop user-friendly devices for the elderly to increase the popularity of virtual nursing homes and smart care devices. In addition, digital literacy education for older adults should be constructed to improve digital skills and aid them in crossing the digital divide [88].

(3) Using conformist mentality for advocacy and expanding social influence

Social influence in this study refers to the perceived status of virtual nursing homes by others and the publicity of the external environment among seniors. Unlike the development process in Western countries, the virtual nursing home in China is a product promoted by government policy. This promotion can enhance older people’s cognition and increase their willingness to use by enhancing government advocacy when older people are confused by facing new things [89]. To increase participation, the government should play a leading role in public affairs as an authority, integrate the efforts of all parties, link up with society and the community [90], promote the construction achievements of virtual nursing homes using multiple channels, and, at the same time, expand the scope of publicity to the 20–60 age group and call on the whole society to create a positive atmosphere to face the aging. Collectivist values, a fundamental element of Chinese culture, play a vital role here, as it becomes a widespread societal trend to positively embrace aging, and individuals are likely to seek alignment with the collective. To achieve this, they tend to be more inclined to observe and follow the actions of others and to heed authoritative advice. Furthermore, the platform should utilize the reinforcing effect of a conformist mentality on social influence by inviting local opinion leaders among the elderly to review the platform’s services so that the virtual nursing home can be promoted with the interpersonal network of the elderly.

(4) Improving services and guiding positive attitudes of users

The elderly care more about risk loss than receiving; hence, as a matter that most elderly do not understand, if the services provided by virtual nursing homes are not performed properly and bring losses to older people, it will cause resistance and then negative attitudes among older people. To guide the positive attitude of seniors, virtual nursing home platforms need to improve the service quality and innovate the service methods, like time banks [91], to motivate seniors to use the virtual nursing home service platforms.

### 6.4. Limitations and Future Research

Although this study has some research implications, it still comes with some limitations. First, in terms of model design, this study focused on the influence of drivers on the behavioral intention to use but did not identify the influence of risk perception factors on the behavior of the elderly. This represents a direction for future research, and future research will include more suitable variables to expand the model. Second, considering that virtual nursing homes are not yet popular across the country, this study was conducted in a spatially developed region with a virtual nursing home service platform, and the sample population was selected to include some users of the platform who are over 60 years old who have used virtual nursing home services. In addition, in the Chinese context, the largest group of baby boomers born in (1963–1973) will become elderly in a short period, while the impact of such groups on the results is not taken into account in this study.

To sum up, in future research, we will expand the selection of regions and increase the sample size by setting the survey population to over 55 years old to improve the accuracy of the analysis results. Finally, for the research issue regarding the mechanism driving the virtual nursing home, this study was analyzed from the perspective of the elderly. However, the behavior of other subjects such as the government and enterprises also affects the willingness of the elderly to use the virtual nursing home. Thus, to complete a more comprehensive study on the driving force for using the virtual nursing home, we will focus on the influence of the other subjects in future research.

## 7. Conclusions

The purpose of this study is to measure the driving factors affecting behavioral intention to use virtual nursing homes among the elderly. We firstly modified the UTAUT model to make it more consistent with the theme of virtual nursing homes in the Chinese context. Then, we confirmed the driving effect of PE, EE, and SI on the behavioral intention of virtual nursing homes, using the attitude toward use as the mediator variable of PE and EE driving the intention. The results showed that attitude significantly affected the impact of PE and EE on the intention. This study also included the conformist mentality under collectivist values in the model, revealing its strengthening effect on the social impact. The research results also contributed by enriching the theory of virtual nursing homes and the expansion of the application scenarios of the UTAUT model.

## Figures and Tables

**Table 1:** UTAUT (extended/modified) in the “Internet+” context.

Ref.	Authors	Objectives	Extended/Modified Constructs	Sample	Key Factors Affecting the Adoption
[19]	Cimperman et al. (2016)	To predict the factors affecting older users’ acceptance of home telehealth services (HTSs)	Computer anxiety, doctor’s opinion, and perceived security	Older persons aged 50 years and above (n = 400)	Performance expectancy, effort expectancy, facilitating conditions, perceived security, computer anxiety, and doctor’s opinion
[20]	Yamin & Alyoubi (2020)	To explore the utilization of wireless sensor network applications (WSN) for telemedicine during the COVID-19 pandemic	Task technology fit, awareness, and self-efficacy	All target audience (n = 348)	Performance expectancy, social influence, effort expectancy, facilitating conditions, task technology fit, awareness, and self-efficacy
[22]	Chong et al. (2015)	To predict the adoption of RFID	Individual differences and demographic characteristics	Physicians and nurses (n = 252)	Individual differences are better predictors of use than variables derived from the UTAUT
[23]	Jewer (2018)	To develop and test a modified model for patient acceptance and utilization of an emergency department (ED) wait times website	Remove voluntary	Patients (n = 118)	Facilitating conditions
[25]	Alam et al. (2020)	To examine the factors affecting the adoption of mHealth services	Perceived reliability and price value	Generation Y aged 18 to 40 (n = 296)	Performance expectancy, social influence, facilitating conditions, and perceived reliability
[42]	Maswadi et al. (2022)	To examine the determining factors of elderly behavioral intention (BI) to use smart home technologies (SHT)	Cultural influence, technology awareness, attitude, education, and region	Elderly people (n = 486)	Cultural influence and technology awareness
[43]	Alkhowaiter (2022)	To explore user attitudes toward mobile payments and their behavioral intentions	Trust, attitude, and religiosity	All target audience (n = 510)	Performance expectancy, facilitating conditions, and trust

**Table 2:** Sample description.

Variables	Description	Frequency (N = 200)	Percent (%)
Gender	Male	86	43.0
	Female	114	57.0
Age	60–64	65	32.5
	65–69	60	30.0
	70–74	30	15.0
	75–79	27	13.5
	80 and above	18	9.0
Formal education	No formal education	31	15.5
	Elementary	44	22.0
	Junior	113	56.2
	High school and above	12	6.0
Physical condition	Very bad	29	14.5
	Bad	56	28.0
	Relatively good	67	33.5
	Healthy	48	24.0
Dwelling type	Live alone	129	64.5
	Live with family	71	35.5
Annual income (CNY)	Below 30,000	139	69.5
	30,000–50,000	49	24.5
	More than 50,000	12	6.0

**Table 3:** Construct reliability results.

Type of Variable	Construct	Item	Factor Loading	Cronbach’s α	AVE	CR	Overall Cronbach’s α
Independent variables	Performance expectancy (PE)	PE1	0.835	0.955	0.737	0.856	0.949
		PE2	0.810				
		PE3	0.805				
		PE4	0.822				
		PE5	0.840				
		PE6	0.829				
		PE7	0.717				
	Effort expectancy (EE)	EE1	0.770	0.910	0.717	0.810	
		EE2	0.742				
		EE3	0.727				
		EE4	0.747				
	Facilitating conditions (FCs)	FC1	0.786	0.887	0.626	0.838	
		FC2	0.831				
		FC3	0.836				
	Social influence (SI)	SI1	0.815	0.902	0.598	0.912	
		SI2	0.836				
		SI3	0.823				
		SI4	0.779				
Mediator	Attitude toward use (ATU)	ATU1	0.834	0.912	0.623	0.843	
		ATU2	0.808				
		ATU3	0.842				
		ATU4	0.820				
Moderator	Conformist mentality (CM)	CM1	0.778	0.919	0.599	0.822	
		CM2	0.816				
		CM3	0.825				
Dependent variable	Behavioral intention (BI)	BI1	0.815	0.830	0.627	0.824	
		BI2	0.728				
		BI3	0.828				

Note: AVE = average variance extracted, CR = composite reliability.

**Table 4:** Pearson correlation coefficient and the square root of AVE.

	PE	EE	FC	SI	ATU	CM	UI
PE	**0.858**						
EE	0.514 **	**0.847**					
FC	0.507 **	0.642 **	**0.791**				
SI	0.611 **	0.641 **	0.449 **	**0.773**			
ATU	0.401 **	0.540 **	0.544 **	0.427 **	**0.789**		
CM	0.194 **	0.110 **	0.088	0.135	0.188	**0.774**	
BI	0.536 **	0.614 ***	0.497 **	0.423 **	0.489 **	0.187 **	**0.792**

Note: Values (bold) in the diagonal area are the square roots of the AVE; significance was determined at = *** *p* < 0.001; ** *p* < 0.01.

**Table 5:** Model fitting indicator results.

Index	χ^2^/df	RMSEA	CFI	NFI	NNFI	TLI	IFI
Reference values	<3	<0.08	>0.9	>0.9	>0.9	>0.9	>0.9
Analysis result values	1.501	0.072	0.957	0.927	0.945	0.945	0.957
Model adaptation judgment	Yes	Yes	Yes	Yes	Yes	Yes	Yes

**Table 6:** Examination of the main and mediating effect.

Variables	UI					ATU	
	Model 1	Model 2	Model 3	Model 4	Model 5	Model 6	Model 7
Control variables							
Age	−0.052	−0.030	−0.012	−0.036	−0.041	−0.082	−0.028
Physical condition	0.014	−0.023	0.011	−0.005	−0.010	0.006	−0.030
Dwelling type	−0.053	−0.047	−0.036	−0.034	−0.035	−0.037	−0.004
Annual income	0.098	0.054	0.086	0.062	0.060	0.026	−0.014
Independent variables							
PE		0.169 **		0.294 ***	0.235 ***		0.846 ***
EE		0.334 ***		0.459 ***	0.377 ***		0.106 ***
FC		−0.009					
SI		0.318 ***					
Mediator							
ATU			0.485 ***		0.166 ***		
F	0.764	23.816 ***	12.823 ***	26.288 ***	22.867 ***	0.443	148.320 ***
R^2^	0.015	0.499	0.248	0.450	0.555	0.009	0.822

Note: The table shows the β values; significance was determined at = *** *p* < 0.001; ** *p* < 0.01.

**Table 7:** Moderating effects test.

Variables	BI					
	Model 8		Model 9		Model 10	
	β	T	β	T	β	T
Age	−0.052	−0.720	−0.002	−0.034	−0.010	−0.190
Physical condition	0.014	0.197	−0.046	−0.855	−0.040	−0.772
Dwelling type	−0.053	−0.746	−0.046	−0.862	−0.049	−0.941
Annual income	0.098	1.368	0.042	0.788	0.040	0.767
SI			0.660	12.390 ***	0.660	12.607 ***
CM			0.270	5.055 ***	0.250	4.711 ***
SI × CM					0.145	2.774 **
F	0.764		28.359 ***		26.250 ***	
R^2^	0.015		0.459		0.489	

Note: Significance was determined at = *** *p* < 0.001; ** *p* < 0.01.

## Data Availability

Not applicable.

## Appendix A. Measurement Items

**Constructs**	**Items**	**Measures**	**Sources**
Performance expectancy (PE)	PE1	The virtual nursing home service platform effectively addresses my basic physiologic needs	[70]
	PE2	The virtual nursing home service platform can sufficiently guarantee my health and security	
	PE3	The virtual nursing home service platform to adequately secure my property	
	PE4	The virtual nursing home service platform can expand my communicative circle	
	PE5	I find virtual nursing home service platform can enhance contact with young adults, especially with my children	
	PE6	The virtual nursing home service platform is very helpful to me	
	PE7	The virtual nursing home service platform enriches my life with spiritual pleasure	
Effort Expectancy (EE)	EE1	The operational approach of virtual nursing home service platforms is easy to learn	[38,71]
	EE2	I can skillfully use the virtual nursing home service platform when I need services	
	EE3	The virtual nursing home service platform enables timely updates to my health data and service information	
	EE4	The design of virtual nursing home service platforms and relevant smart devices meets my usage habits	
Facilitating conditions (FCs)	FC1	The virtual nursing home service platform provides me with information on service standards	[38,71]
	FC2	The virtual nursing home service platform provides me with feedback mechanisms to evaluate	
	FC3	I can easily get help from the staff when I have difficulties using the Virtual nursing home service platform	
Social influence (SI)	SI1	Friends on my side suggested that I try to use a virtual nursing home service platforms	[59,71]
	SI2	Many of the elderly living around are using virtual nursing home service platforms	
	SI3	The community often organizes events and builds an atmosphere to promote virtual nursing home service platforms	
	SI4	The policies have been developed by the government to guide me to use virtual nursing home service platforms	
Attitude toward use (ATU)	ATU1	Using virtual nursing home service platforms is a wise idea	[72]
	ATU2	Using virtual nursing home service platforms will make life more comfortable	
	ATU3	I am satisfied with the services of virtual nursing homes	
	ATU4	I feel that virtual nursing homes will later become the primary way of elderly care	
Conformist mentality (CM)	CM1	If I am aware that the authoritative body encourages the use of virtual nursing home platforms, it would motivate me to try them	[65,73,74]
	CM2	If my friends around me are accepting a virtual nursing home, I would be open to considering it, even if it was not my initial idea	
	CM3	The more people in my surrounding community using virtual nursing home services, the more I want to use	
Behavioral intention (BI)	BI1	I will always try to use virtual nursing home service platform in my daily life	[75]
	BI2	I plan to use virtual nursing home service platform frequently	
	BI3	I plan to recommend virtual nursing home service platform to those around	
